# Permanent iodine-125 brachytherapy for patients with progressive or recurrent high-grade gliomas

**DOI:** 10.1186/s12885-020-07086-8

**Published:** 2020-06-24

**Authors:** Congxiao Wang, Shifeng Liu, Lijing Peng, Kaixian Zhang, Wei Li, Hao Zhang, Ying Luan, Peishun Li, Xiaokun Hu

**Affiliations:** 1 Department of the Interventional Medical Center, the Affiliated Hospital of Qingdao University, #1677 Wutaishan Road, Qingdao, 266000 Shandong China; 2 Department of Clinical Laboratory, the Affiliated Hospital of Qingdao University, Qingdao, China; 3 Department of Oncology, Tengzhou Central People’s Hospital, Zaozhuang, 277500 Shandon China; 4 Jiangsu Key Laboratory of Molecular and Functional Imaging, Department of Radiology, Zhongda Hospital, Medical School of Southeast University, Nanjing, 210009 China

**Keywords:** Progressive or recurrent high-grade gliomas, Iodine-125 brachytherapy, Radiotherapy, Chemotherapy

## Abstract

**Background:**

The prognosis of patients with progressive or recurrent high-grade gliomas (HGGs) after surgery remains poor. Iodine-125 brachytherapy is emerging as a salvage method for the treatment of gliomas. This study aimed to investigate whether permanent iodine-125 brachytherapy could be used as an effective therapeutic method even without radiotherapy and/or chemotherapy for progressive or recurrent HGG after gross total resection.

**Methods:**

Between March 2004 and August 2016, 58 patients with progressive or recurrent HGG after gross total resection were included in this study. Twenty-nine patients underwent radiotherapy and/or chemotherapy and then permanent iodine-125 brachytherapy (SRCI group). Twenty-nine patients underwent permanent iodine-125 brachytherapy alone (SI group). Follow-up was carried out at 1, 3, and 6 months and then at 1, 2, 3, and 5 years after iodine-125 implantation. The median overall survival (OS) and progression-free survival (PFS), procedure-related complications and clinical outcomes were evaluated.

**Results:**

No procedure-related fatal events happened. The temporary morbidity rate was 11.9%. The median OS and PFS for patients in the SI group were 22 and 8 months compared with 21 and 7 months in the SRCI group. No significant differences were found. Age and Karnofsky Performance Status (KPS) were independent prognostic factors for OS. Age, KPS and histology were independent prognostic factors for PFS.

**Conclusions:**

Permanent iodine-125 brachytherapy could be used as an effective therapeutic method even without radiotherapy and/or chemotherapy for progressive or recurrent HGG after gross total resection.

## Background

High-grade gliomas (HGGs) are the most common primary brain tumors, and HGG accounts for approximately 2% of all cancers and 70–80% of all brain tumors [1–3]. Gliomas are thought to originate from glial, stem or neuronal precursor cells. According to the WHO criteria, gliomas are histologically classified into four different grades (grade I to grade IV), and grade III and grade IV tumors are the so-called high-grade or malignant gliomas [4]. HGG is one of the most devastating human diseases due to its diffusely infiltrating spread characteristics [5]. Primary tumor resection is the first-line therapy for HGG [4]. However, the prognosis of patients remains poor regardless of aggressive treatment, and most patients die due to the disease or related complications, with a median survival of 9–12 months [4, 6]. Owing to the diffuse and infiltrative characteristics of the tumor, malignant gliomas often recur even when gross total resection has been carried out [4]. The treatment for progressive or recurrent HGG remains a controversial subject. For patients with progressive or recurrent HGG, repeated surgery is usually unfeasible, and radiotherapy and/or chemotherapy are usually chosen but are often not effective options; moreover, their safety also remains a question [7].

Iodine-125 brachytherapy is emerging as a promising therapeutic method for gliomas that promotes the survival of patients [6, 8]. The implantation of iodine-125 seeds is a safe, accurate and effective treatment method with minimal invasion and a consecutive low-dose rate of radiation that places the seeds within the tumor [6, 9], which is different from external beam radiotherapy (EBRT). Recently, iodine-125 brachytherapy has been suggested to be an effective salvage therapy for patients with progressive or recurrent gliomas [7, 10]; however, whether radiotherapy and chemotherapy before iodine-125 brachytherapy is necessary has not been explored.

In this study, we aimed to investigate whether permanent iodine-125 brachytherapy could be used as an effective therapeutic method even without radiotherapy and/or chemotherapy for progressive or recurrent HGG after gross total resection. The advantages will also be explored.

## Methods

### Patient criteria

This study retrospectively analyzed patients from the database of the Affiliated Hospital of Qingdao University in China from Mar. 2004 to Jun. 2017. The study was approved by the Institutional Ethics Review Boards of the Affiliated Hospital of Qingdao University with the requirement of informed consent waived (Reference number: QYFY WZLL 25802). Patients who met the following criteria were included in our study: (a) 16 years of age or older; (b) histologic diagnosis of WHO grade III or IV glioma; (c) contrast-enhancing tumor; (d) relapse of glioma after gross total tumor resection; and (e) availability of adequate laboratory examination information, including hematologic parameters, clotting, hepatic and renal function, etc. Patients were excluded if the tumor involved the brain stem or ependymal surface.

The 58 patients included in this study were divided into two groups. The SI group (surgery+ iodine-125 brachytherapy, *n* = 29) included patients with progressive or recurrent HGG after gross total resection who received permanent iodine-125 brachytherapy. The SRCI group (surgery+ radiotherapy and/or chemotherapy+ iodine-125 brachytherapy, *n* = 29) included patients with progressive or recurrent HGG after gross total resection who received radiotherapy and/or chemotherapy; when relapse was observed, permanent iodine-125 brachytherapy was given.

### Study outcomes

The outcomes observed were median overall survival (OS) and median progression-free survival (PFS). OS was defined as the time from the diagnosis of HGG to the date of death or the last follow-up. PFS was defined as the time from the implantation of iodine-125 seeds to the diagnosis of tumor recurrence or progression. Additionally, univariate analysis (log-rank test, *P* < 0.05) and multivariate analysis (Cox proportional hazards model, P < 0.05) were performed to determine the possible prognostic factors for OS and PFS.

### Treatment plans

All patients with progressive and recurrent HGG had previously underwent gross total resection. For iodine-125 seed implantation, a computerized treatment planning system (TPS; Beijing Astro Technology Ltd. Co., Beijing, China) was used. Based on computed tomography (CT) or magnetic resonance imaging (MRI) images before implantation, the prescribed dose (PD) of 100–150 Gy was administered. The planning target volume (PTV) consisted of the region of the gross target volume plus a 10 mm margin in all three dimensions defined by CT scan. The insertion site, path, and direction of the needles were decided, and seeds were designed 5–10 mm apart. The isodose curve distribution and dose volume histogram (DVH) were plotted, and the D90, D100, V200, V100, and V90 were calculated. After the surgery, the TPS was used to confirm the rationality of the seed distribution.

### Iodine-125 implantation

The patients were securely positioned on the CT scan bed with a negative pressure vacuum pad. They were locally anesthetized with 2% lidocaine, and an incision was made with a blade on the scalp. Holes with diameters of 2 mm–4 mm were made with an electric cranial drill. A dynamic CT scan was carried out before the implantation to show the boundary of the gliomas. Iodine-125 seeds (diameter of 0.8 mm, length of 4.5 mm, half value of 0.025 mm in lead, half-life of 59.4 days; Model 7711, Beijing Atom and High Technique Industries, Inc., Beijing, China) were implanted with flat needles; the path of the needles and the distribution of the seeds during the operation were dynamically monitored and rectified with intraoperative CT scans, and dosimetric verification was performed with the TPS during the operation to help with the implantation. The galea aponeurosis and the scalp were sutured after implantation to prevent the leakage of cerebrospinal fluid. The whole surgery lasted for 1–2 h altogether. After implantation, vital signs were monitored, and the patients were required to be inactive for 24 h and were routinely treated with dehydration medications for 7–14 days.

### Patient evaluation

The status of the patients was evaluated before the surgery of iodine-125 seed implantation and immediately when they were discharged from the hospital. Complications after implantation were also recorded. The follow-ups were carried out at 1, 3, and 6 months and then at 1, 2, 3, and 5 years after seed implantation, mainly via telephone interviews. Headache, nausea, vomiting, vertigo, myodynamics, vision, hearing, aphasia, epilepsy and sensory function were evaluated before and after seed implantation. OS and PFS were compared between the two groups. Causes of death were recorded.

### Statistical methods

The last follow-up was on 31 Jul. 2017. The data were analyzed using SPSS (Version 18.0, IBM, NY, USA). Categorical variables are presented as frequencies and percentages, and continuous variables are presented as medians and ranges. The baseline characteristics of the patients in the two groups were compared with χ2 or Fisher exact tests for categorical variables or two-tailed Student’s t-test for continuous variables. OS and PFS were calculated with the Kaplan-Meier method. The log-rank test was used for the univariate analysis, and all variables with *P* ≤ 0.1 or those (*p* > 0.1) thought to be clinically important were included in the multivariate analysis. A Cox proportional hazards model was used for multivariate analysis. *P* < 0.05 was considered statistically significant.

## Results

### Patient characteristics

Table 1 shows the baseline characteristics of the 58 patients with progressive or recurrent HGG after gross total resection in this study. All the patients in the SRCI group were treated with EBRT (median, 60 Gy; range, 34–70 Gy). Of the 29 patients in the SRCI group, 11 underwent adjuvant chemotherapy with temozolomide. More patients had epilepsy in the SI group (22 of 29, 75.9%) than in the SRCI group (11 of 29, 37.9%). No significant differences were found in the other symptoms before seed implantation, such as headache, nausea, vomiting, vertigo, myodynamics, vision, hearing, aphasia and sensory function. The other variables were not significantly different between the two groups.

**Table 1:** Patients and Implantation Characteristics

Characteristics	SI(n = 29)	SRCI(n = 29)	p Value^a^
**Age (yrs)**			0.637
Median (Range)	46 (20–73)	46 (16–70)	
**Sex**			0.588
Male	17 (58.6)	19 (65.5)	
Female	12 (41.4)	10 (34.5)	
**KPS**	22 (75.9)		
Median (Range)	80 (50–80)	80 (50–90)	0.401
90–100	11 (37.9)	8 (27.6)	
≤ 80	18 (62.1)	21 (72.4)	
**Extent of Resection**			
Gross total resection	29 (100)	29 (100)	
Partial resection	0 (0)	0 (0)	
Biopsy	0 (0)	0 (0)	
**ERBT dose (Gy)**			
Median (Range)	/	60 (34-70Gy)	
**Adjuvant chemotherapy**	/	11 (37.9)	
**Tumor position**			
Frontal lobe	6 (20.7)	11 (37.9)	
Occipital lobe	1 (3.5)	0 (0)	
Parietal lobe	4 (13.8)	0 (0)	
Temporal lobe	9 (31.0)	6 (20.7)	
multi-lobe	9 (31.0)	12 (41.4)	
**Tumor location**			
Left hemisphere	16 (55.2)	13 (44.8)	
Right hemisphere	11 (37.9)	16 (55.1)	
Both hemisphere	2 (6.9)	0 (0)	
**Tumor Diameter**			0.588
≥ 5 cm	12 (41.4)	10 (34.5)	
< 5 cm	17 (58.6)	19 (65.5)	
**Histology**			0.753
WHO III	7 (24.1)	6 (20.7)	
WHO IV	22 (75.9)	23 (79.3)	
**No. of seeds**			0.368
Median (Range)	45 (16–150)	40 (20–98)	
**PD (Gy)**	110 (100–150)	110 (100–150)	0.655
**Symptoms prior to implantation**			
Headace	27 (93.1)	27 (93.1)	> 0.999
Nausea	22 (75.9)	18 (62.1)	0.256
Vomit	14 (48.3)	12 (41.4)	0.597
Vertigo	6 (20.7)	9 (31.0)	0.368
Myodynamic			0.387
≥ grade 4	22 (75.9)	19 (65.5)	
< grade 4	7 (24.1)	10 (34.5)	
Vision	6 (20.7)	10 (34.5)	0.240
Hearing	5 (17.2)	3 (10.3)	0.446
Aphasia	6 (20.7)	4 (13.8)	0.487
Epilepsy	22 (75.9)	11 (37.9)	0.004
Sensory	3 (10.3)	5 (17.2)	0.446

Note-Date in parentheses are percentages unless indicated

KPS, Karnofsky Perfomance Scale; EBRT, external beam radiotherapy

^a^χ2 test or Student’s t-test tests were used

### Iodine-125 seed implantation

**Fig. 1:**
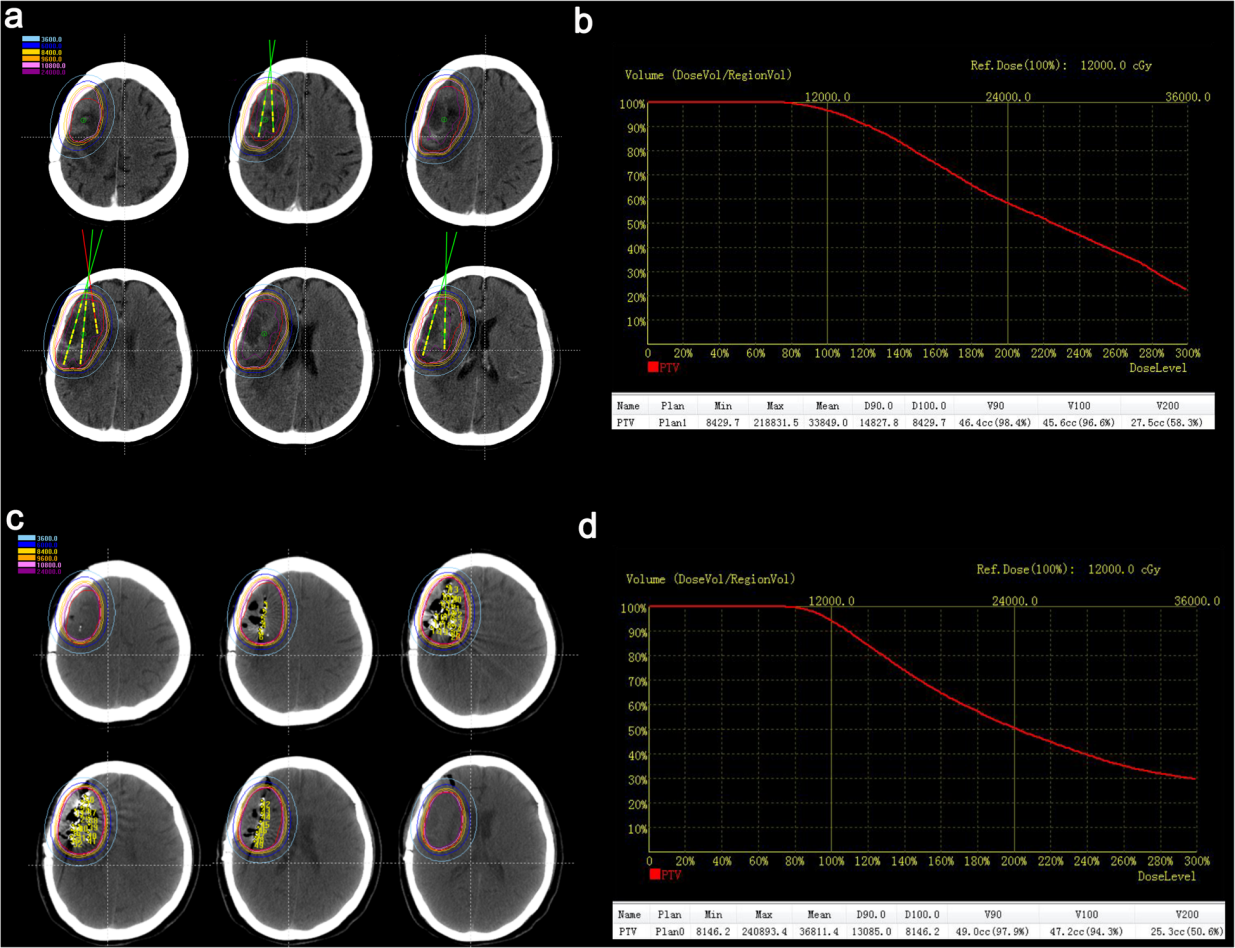
Treatment planning and verification. (a) The planning target volume (PTV) is outlined with a red line, and the dose distribution is outlined with different colored lines. Additionally, a needle path was designed before the operation with the computerized treatment planning system. (b) Dose volume histogram (DVH) of PTV preoperation. (c) Verification of the dose distribution postsurgery. (d) DVH of PTV postoperation

Before iodine-125 seed implantation, an implantation plan was made with a computerized TPS. The needle path, seed distribution, isodose curve, and DVH are shown in Fig. 1a-b. After the implantation, the plan was verified, as shown in Fig. 1c-d.

### Perioperative mortality and morbidity

No surgery-related fatal events happened (perioperative mortality rate, 0%). The temporary morbidity rate was 11.9%. Cerebral hemorrhage happened in 4 patients (6.8%), and 3 patients had heavier brain edema (5.1%). The patients with heavier brain edema improved a few months after the implantation with dehydration treatment, and self-absorption of the cerebral hemorrhage was almost complete approximately 15 days with the conservation treatment.

### Survival analysis

**Fig. 2:**
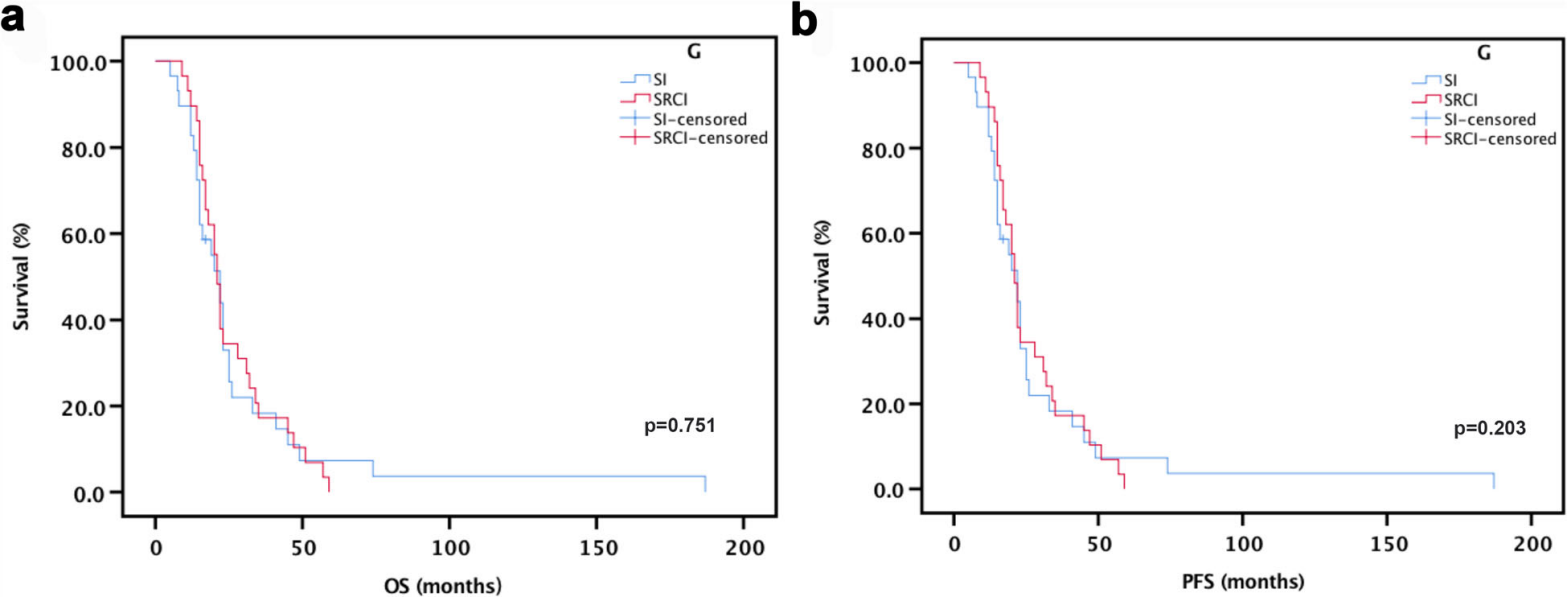
Kaplan-Meier analysis of OS and PFS (a) The median OS was 22 months in the SI group compared with 21 months in the SRCI group (p = 0.751). (b) The median PFS was 8 months in the SI group compared with 7 months in the SRCI group (p = 0.203)

The median OS of the patients was 22.0 months (95% confidence interval (CI): 14.50–29.50 months) in the SI group and 21.0 months (95% CI: 18.89–23.11 months) in the SRCI group. The median PFS of the patients was 8.0 months (95% CI: 5.47–10.53 months) in the SI group and 7 months (95% CI: 4.74–9.26 months) in the SRCI group. No significant differences were found between the two groups in OS (*p* = 0.751) or PFS (*p* = 0.203) (Fig. 2a-b).

One patient lost contact, and the other 57 patients died during our follow-up. Four patients in the SI group survived more than 36 months, with the longest survival time being 187 months. Five patients in the SRCI group survived more than 36 months, with the longest survival time being 59 months.

### Prognostic factors

To further investigate the factors related to the survival of patients, we first performed univariate analysis of covariates, and the results showed that age and Karnofsky Performance Status (KPS) were significant for OS. Age, KPS, tumor diameter, and nausea were significant for PFS (Table 2). Factors with a *p* value≤0.1 and those thought to be clinically significant were included in the multivariate analysis. The results showed that age and KPS were independent prognostic factors for OS. Age, KPS and histology were independent prognostic factors for PFS (Table 3).

**Table 2:** Univariate analysis for OS and PFS

Univariate Analysis				
	OS		PFS	
**Characteristics**	**Median (mo)**	**p Values**^b^	**Median (mo)**	**p Values**^b^
**Group**		0.751		0.203
SI	22 (14.50–29.50)		8 (5.47–10.53)	
SRCI	21 (18.89–23.11)		7 (4.74–9.26)	
**Age**		0.006		0.029
≥ 60	22 (20.36–23.64)		8 (6.81–9.19)	
< 60	17 (11.82–22.18)		4 (2.06–5.94)	
**KPS**		< 0.001		< 0.001
90–100	35 (23.63–46.38)		12 (8.80–15.20)	
≤ 80	16 (14.17–17.84)		6 (5.14–6.86)	
**Tumor Position**				0.074
Frontal lobe			10 (7.31–12.69)	
Occipital lobe			13	
Parietal lobe			8 (0.00–28.58)	
Temporal lobe			7 (4.30–9.70)	
multi-lobe			6 (5.32–6.68)	
**tumor diameter**				0.015
≥ 5 cm			9 (6.99–11.01)	
< 5 cm			6 (5.24–6.76)	
**Vertigo**				0.074
NO			8 (6.70–9.30)	
Yes			5 (3.64–6.36)	
**Nausea**				0.040
No			9 (6.06–11.94)	
Yes			6 (4.36–7.64)	
**Vomit**				0.078
No			8 (6.38–9.62)	
Yes			6 (4.33–7.67)	

Note.-Date in parentheses are 95% CI

^b^Log-rank test was used

**Table 3:** Multivariate analysis for OS and PFS

Multivariate Analysis				
Characteristics	OS		PFS	
	Hazard Ratio	p Values^c^	Hazard Ratio	p Values^c^
**Age**		0.016		< 0.001
≥ 60	1		1	
< 60	2.70 (1.21–6.03)		4.96 (2.10–11.7)	
**KPS**		< 0.001		0.047
90–100	1		1	
≤ 80	4.42 (1.94–10.07)		2.92 (1.40–6.09)	
**Histology**				0.008
WHO 3			1	
WHO 4			3.99 (1.43–11.14)	

Note.-Date in parentheses are 95% CI

^c^Cox proportional hazards regression analysis was used

### Follow-up after Iodine-125 implantation

Quality of life was assessed with KPS at different time points. The mean pretreatment KPS score was 80 (range 50–90) in the SI group and 80 (range 50–90) in the SRCI group. No significant differences were found between the groups (*p* = 0.401). The median KPS scores of patients at the time of discharge from the hospital and those who survived for 1 month, 3 months, 6 months, 1 year, 2 years, and 3 years in the SI group were 80 (range 60–100), 80 (range 40–100), 80 (range 30–100), 80 (range 50–100), 90 (range 60–100), 70 (range 50–90), and 70 (range 50–90), and the median KPS scores of patients at the time of discharge from the hospital and those who survived for 1 month, 3 months, 6 months, 1 year, and 2 years in the SRCI group were 70 (range 40–100), 80 (range 40–100), 70 (range 30–100), 70 (range 30–100), 70 (range 50–100), and 70 (range 70–80), respectively. In the SI group, 22 patients died due to brain hernia, 2 patients died due to brain edema, and 5 patients died of unknown causes. In the SRCI group, patients died due to brain hernia (20 of 30 patients), brain edema (1 of 30 patients), leukoencephalopathy (5 of 30 patients), cerebral hemorrhage (1 of 30 patients), pulmonary infection (1 of 30 patients), suicide (1 of 30 patients), and multiple intracranial disease (1 of 30 patients).

## Discussion

HGG is a disease that mainly grows from the site of origin and can spread throughout the brain. It can invade the adjacent brain to some extent, which is invisible to the naked eye or in imaging examination, thus preventing complete oncological resection and promoting resistance to radiotherapy and chemotherapy [11]. For most patients with HGG, surgery is the first-line choice; however, it is hard for neurosurgeons to determine the extent of resection. Thus, relapses of HGG constitute the setback for surgeries [4, 12], which is always a difficult subject for doctors.

Therapies for patients with progressive or recurrent HGG remain a controversial subject. Most patients have already lost the chance for repeated surgery after glioma relapse, and radiotherapy and/or chemotherapy may be the only treatment options available. However, not all patients accept radiotherapy and/or chemotherapy treatment due to the efficacy, time, costs, side effects, etc. The median OS of patients with glioblastoma after surgery treated with radiotherapy and adjuvant temozolomide was only 14.6 months, and for radiology alone, it was 12.1 months [13]. Usually, patients face increased intracranial pressure early after radiotherapy as well as late radiation effects, such as true radionecrosis, leukoencephalopathy syndrome, lacunar infarcts, brain parenchymal calcifications, Moyamoya syndrome, telangiectasias, and enhancing whiter matter abnormalities [13, 14]. Moreover, attempts to improve the survival of HGG patients with increased radiation doses have failed [15].

Iodine-125 seeds are emerging as a safe, effective and minimally invasive method applied in various tumors [16–20]. Several studies have proven that iodine-125 implantation could improve the survival of patients with gliomas [5, 8–10, 21–23]. Especially in China, as more people become aware of this therapeutic method, they tend to prefer it because it is minimally invasive. Iodine-125 seeds are implanted accurately into the exact site of the tumor, which could give a much higher radiation dose and cover a larger radiation area on the oncological border with confirmed safety. Additionally, with permanent implantation, iodine-125 seeds could play continuous roles within the tumor, with the continuous release of low-dose γ rays, which is different from EBRT; iodine-125 brachytherapy administers a higher PD and therapeutic target [24]. Iodine-125 implantation has mostly been used as a salvage therapy after radiotherapy and/or chemotherapy for progressive or recurrent patients with gliomas. However, no evidence has shown whether iodine-125 brachytherapy could be used as a primary therapeutic method for patients with progressive or recurrent HGG after gross total resection. Moreover, whether the survival of patients who received iodine-125 seed implantation without radiotherapy and/or chemotherapy is affected needs to be explored.

In this study, we analyzed 58 patients from 16 hospitals with progressive or recurrent HGG after gross total resection. No significant differences were found between the two groups in OS or PFS. These results indicated that patients may undergo iodine-125 implantation after gross total resection without radiotherapy and/or adjuvant chemotherapy, since it had no effects on their survival or on the relapse of glioma. Based on our experiences, the border of the PTV in this study was 10 mm beyond the gross target volume (GTV), different from the previously reported 5 mm [6], while safety and efficacy were confirmed.

The KPS scores of patients remained stable before and after implantation within 1 year. Most patients died 1 year after the implantation of iodine-125 seeds; thus, the follow-up KPS score 1 year after iodine-125 implantation did not reflect the exact quality of life of the patients.

In contrast to that in other previous studies [6, 8–10], we permanently implanted iodine-125 seeds with a flat needle, effectively avoiding damage to brain vessels and tissues. Safety was confirmed, no surgery-related fatal events happened, and the temporary morbidity rate was 11.9% perioperatively. The condition of patients with heavier brain edema improved a few months after the implantation with dehydration treatment, and self-absorption of the cerebral hemorrhage was almost complete approximately 15 days with the conservation treatment. Based on our experience with more than 400 cases of iodine-125 implantation into brain tumors, we also included 30 patients with tumor diameters beyond 5 cm in this study. Heavier cerebral edema happened in 2 of the 30 patients with tumor diameters beyond 5 cm, and cerebral hemorrhage occurred in 2 of the 30 patients with tumor diameters beyond 5 cm. The complications improved days later with conservation treatment. Thus, we believe that the implantation of iodine-125 seeds in patients with progressive or recurrent HGG with tumor diameters beyond 5 cm is safe. In the present study, both age and KPS were independent prognostic factors for OS and PFS, which was in line with the findings of previous studies [10, 25].

The results indicated that patients who were younger or had higher KPS scores survived for a longer time after multimodality therapy. Moreover, histology was thought to be an independent prognostic factor for PFS. Glioma relapse happened more often in patients who were diagnosed with WHO grade IV gliomas. Additionally, there are limitations in this study. The sample size analyzed in this study was not large enough. Only patients who underwent gross total resection were analyzed, and patients who underwent subtotal resection or biopsy were not included, so further studies are needed.

## Conclusions

Altogether, the present study showed that allowing for costs, fees and side effects, for progressive or recurrent HGG patients after gross total resection who have lost the chance for repeat surgery, iodine-125 brachytherapy could be an effective therapeutic method even without radiotherapy and/or chemotherapy.

## Data Availability

The datasets used and/or analysed during the current study are available from the corresponding author on reasonable request.

## Abbreviations

HGGs: High-grade gliomas
OS: Overall survival
PFS: Progression-free survival
KPS: Karnofsky Performance Status
EBRT: External beam radiotherapy
CT: Computed tomography
MRI: Magnetic resonance imaging
PD: Prescribed dose
GTV: Gross target volume
