# Correction to: Permanent iodine-125 brachytherapy for patients with progressive or recurrent high-grade gliomas

**DOI:** 10.1186/s12885-020-07246-w

**Published:** 2020-08-07

**Authors:** Congxiao Wang, Shifeng Liu, Lijing Peng, Kaixian Zhang, Wei Li, Hao Zhang, Ying Luan, Peishun Li, Xiaokun Hu

**Affiliations:** 1 Department of the Interventional Medical Center, the Affiliated Hospital of Qingdao University, #1677 Wutaishan Road, Qingdao, 266000 Shandong China; 2 Department of Clinical Laboratory, the Affiliated Hospital of Qingdao University, Qingdao, China; 3 Department of Oncology, Tengzhou Central People’s Hospital, Zaozhuang, 277500 Shandon China; 4 Department of Radiology, Zhongda Hospital, Medical School of Southeast University, Jiangsu Key Laboratory of Molecular and Functional Imaging, Nanjing, 210009 China

**Correction to: BMC Cancer 20, 591 (2020)**

**https://doi.org/10.1186/s12885-020-07086-8**

Following publication of the original article [1], the authors identified an error in Fig. 2. The corrected Fig. 2 is given below.

**Fig. 2:**
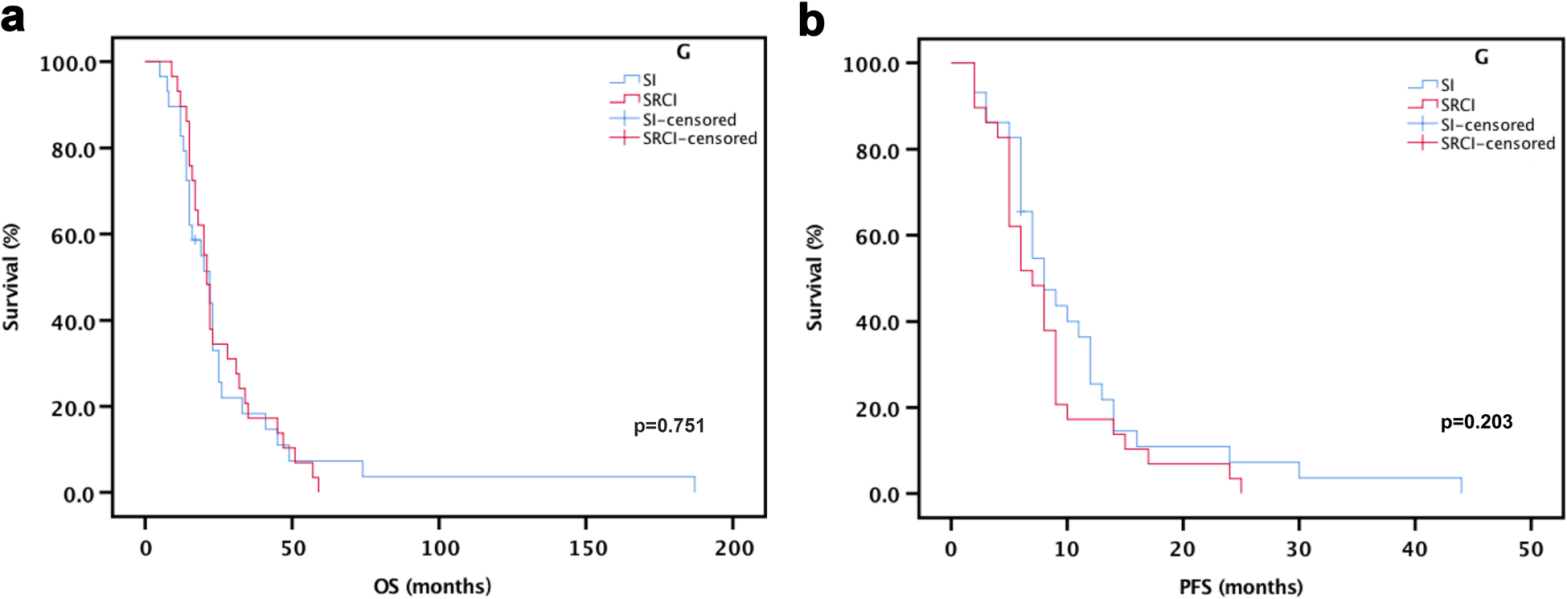
Kaplan-Meier analysis of OS and PFS **a** The median OS was 22 months in the SI group compared with 21 months in the SRCI group (*p* = 0.751). **b** The median PFS was 8 months in the SI group compared with 7 months in the SRCI group (*p* = 0.203)
